# Supplementary material for: Alpha Radiation as a Way to Target Heterochromatic and Gamma Radiation-Exposed Breast Cancer Cells
Source: Cells. 2020 May 8;9(5):1165. doi: 10.3390/cells9051165 (PMC7291130; doi:10.3390/cells9051165)
Supplement: Supplementary file 1 [file cells-09-01165-s001.pdf]

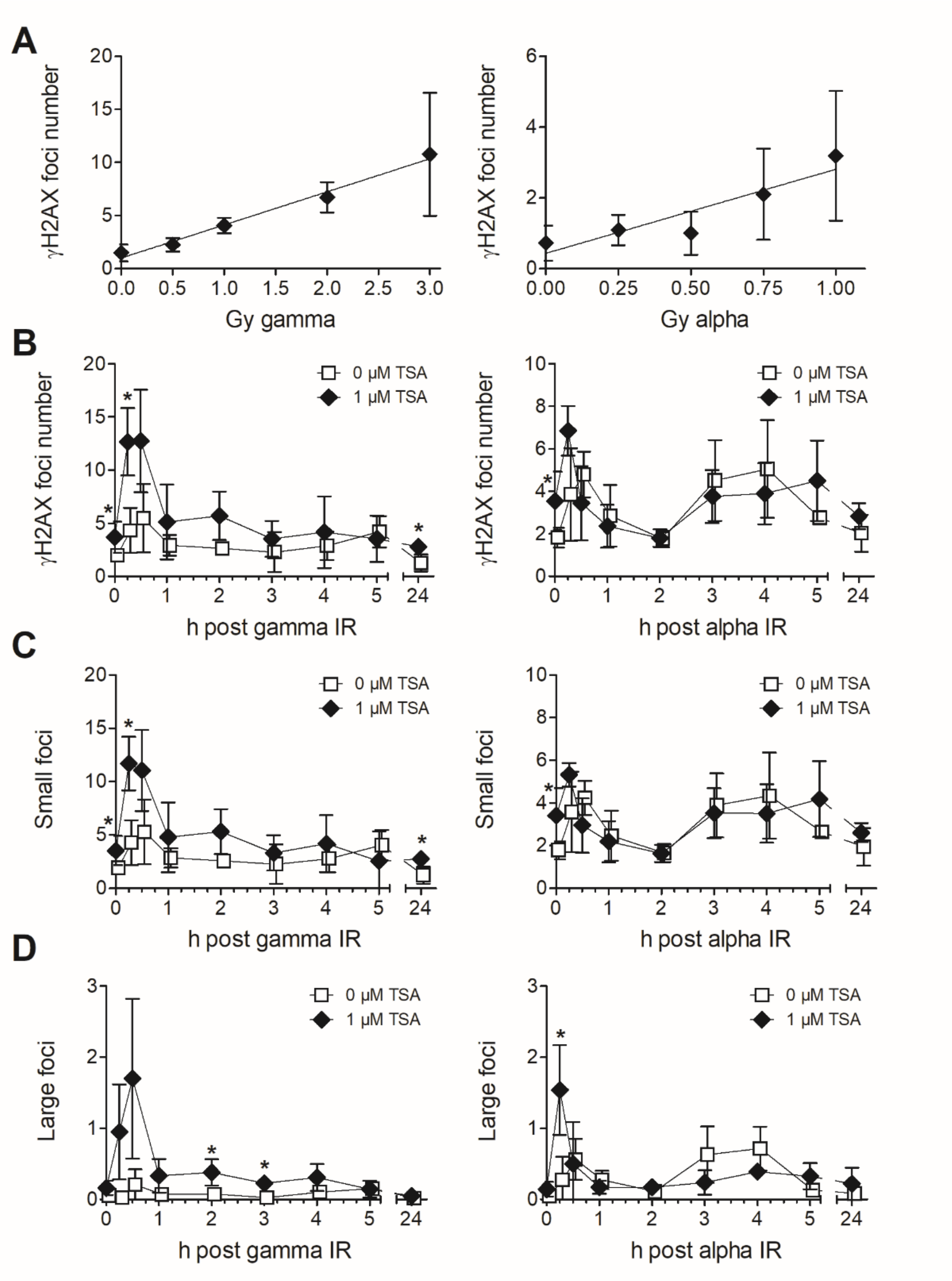

Supplemental Figure S1. Data represent original foci numbers, without subtraction of control values as in Figure 2. (**A**) The dose response of gamma and alpha radiation was evaluated at 30 min post irradiation by analysis of the γH2AX foci number in MDA-MB-231 cells. (**B**) The repair kinetics of γH2AX foci are presented from 15 min up to 24 h post exposure to 2 Gy of gamma or 0.75 Gy of alpha radiation in MDA-MB-231 cells pretreated with 1 μM TSA for 18 h. The numbers of small (**C**) and large (**D**) foci are displayed, using the data from (Figure 2c). *; $p < 0.05$ versus 0 μM TSA.

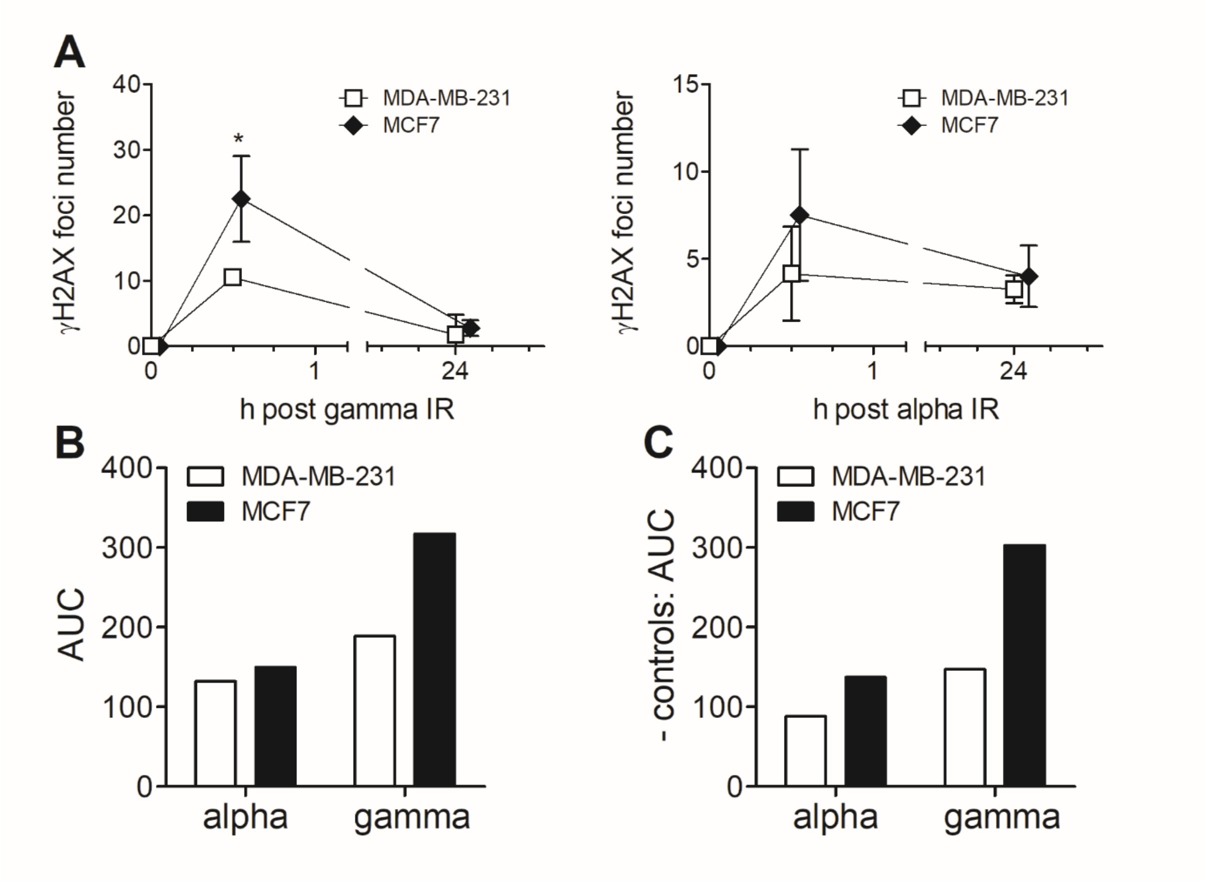

Supplemental Figure S2. (**A**) γH2AX foci numbers (with foci numbers for controls subtracted) are presented at 30 min and 24 h post exposure to 6 Gy of gamma or 2 Gy of alpha radiation in MDA-MB-231 and MCF7 cells. *; $p < 0.05$, versus MDA-MB-231. Areas under the curve (AUC) values are presented for the γH2AX foci numbers from Figure 6 (**B**) as well as the subtracted ones from **A** (**C**).
